# Functionalized Magnetic Bacterial Cellulose Beads as Carrier for Lecitase® Ultra Immobilization

**DOI:** 10.1007/s12010-018-2816-1

**Published:** 2018-06-18

**Authors:** Radosław Drozd, Magdalena Szymańska, Rafał Rakoczy, Adam Junka, Patrycja Szymczyk, Karol Fijałkowski

**Affiliations:** 1 Department of Immunology, Microbiology and Physiological Chemistry, Faculty of Biotechnology and Animal Husbandry, West Pomeranian University of Technology, Szczecin, 45 Piastów Avenue, 71-311 Szczecin, Poland; 2 Faculty of Chemical Technology and Engineering, Institute of Chemical Engineering and Environmental Protection Processes, West Pomeranian University of Technology, Szczecin, 42 Piastów Avenue, 71-065 Szczecin, Poland; 3 Department of Pharmaceutical Microbiology and Parasitology, Medical University of Wroclaw, Borowska 211, 50-556 Wrocław, Poland; 4 Centre for Advanced Manufacturing Technologies, Wroclaw University of Technology , Łukasiewicza 5, 50-370 Wrocław, Poland

**Keywords:** Bacterial cellulose, Modification, Immobilization, Lecitase® Ultra

## Abstract

Bacterial cellulose spheres subjected to amination and inlaid modification with superparamagnetic molecules were analyzed with regard to possibility of their application as an immobilization carrier of Lecitase® Ultra (LU) enzyme. The starting point to obtain the carrier was synthesis of bacterial cellulose spheres performed in shaking cultures of *Komagataeibacter xylinus*. These spheres were subsequently subjected to a multi-stage modification to increase the efficiency of the immobilization process and to separate product from the reaction medium. Maximal yield of Lecitase® Ultra immobilization equaled 70%. It was also found that immobilization process did not affect the pH and LU temperature optimum. Moreover, immobilized enzyme exhibited similar temperature stability profile as its native form. The immobilization process did not significantly affect the enzyme K_M_ value. The immobilized enzyme retained over 70% of its initial activity after 8 cycles of use. The immobilized enzyme displayed good storage stability and retained 80% of its initial activity after 4 weeks at 4 °C. The potential application of such modified cellulose-based carrier may be correlated with lower costs of process thanks to higher enzyme’s reusability in comparison to unbound enzyme. Moreover, data presented in the current study may serve as proof of a concept of cellulose-based carrier utilization for immobilization of enzymes other than LU and of high industrial importance.

**Electronic supplementary material:**

The online version of this article (10.1007/s12010-018-2816-1) contains supplementary material, which is available to authorized users.

## Introduction

Immobilization allows to use enzymes in many industrial branches thanks to the possibility of their repeated use (it lowers costs of new enzymes’ production) and increased stability related with severe for biological macromolecules conditions of reactions, especially in industrial settings [1]. The process of enzymes’ immobilization is referred by some authors as “an art,” and it requires, more than anything else, using a suitable carrier that meets all pre-designed requirements [2, 3]. In the era of searching for environment-friendly technologies, natural biopolymers are gaining more and more recognition [4]. One of such biopolymers, with such unique properties, as high homogeneity or high Young’s modulus, is bacterial cellulose (BC) [5]. Depending on the bacterial culturing conditions, BC takes form of flat membranes (in static cultures), or spheres (when shaking is performed). The BC size, mechanical properties, degree of crystallinity, or polymerization also depends on the culture conditions [6, 7]. Despite many advantages, purified BC lacks specific functional groups that allow to permanently bind enzymes to its fibrils. Interactions between enzyme and BC are result of hydrophobic or hydrogen bond interactions which are susceptible to temperature changes, pH, or ionic strength. The BC matrix for enzyme immobilization may be modified in situ by supplementation of the culture medium with, e.g., carboxymethylcellulose, chitosan, alginate, or lignin derivatives [8–11]. Another way to modify the physicochemical properties of BC is to modify the conditions of drying of BC membranes, which affect its porosity and the ability to adsorb the enzyme [12]. BC can also be modified by introduction of epoxide groups using epichlorohydrine or 1,4-butanediol diglycidyl ether and further amination or oxidization using 2,2,6,6-tetramethylpiperidin-1-oxyl (TEMPO) to introduce carboxyl groups on its surface [13–16]. These treatments considerably increase the possibilities of using BC as enzyme or other active substances’ carrier.

Lipolytic enzymes, despite many years of research on their properties and applicability, still attract great attention thanks to their high biotechnological potential. For example, in the food industry, lipolytic enzymes are increasingly used as replacements or additives to traditional emulsifiers [17, 18]. Lipases have also become one of the most important groups of enzymes used in organic syntheses. The ability to catalyze ester synthesis and transesterification reactions allows the use of this enzyme as biocatalysts in the production of biodegradable polyesters [19, 20]. Thus, methods increasing lipases’ efficiency and operational stability, including enzymes’ immobilization, are constantly developed [21].

The aim of the present study was to analyze the properties of BC spheres obtained from a *Komagataeibacter xylinus* shaking culture, modified with polyethyleneimine and ferromagnetic particles, for use as a carrier for the immobilization of enzymes of lipolytic activity, namely, Lecitase® Ultra.

## Material and Methods

### Materials

An enzymatic preparation displaying activity of phospholipase A and lipase (trade name Lecitase® Ultra; E.C.3.1.1.32, LU, Sigma-Aldrich) was used. *K. xylinus* ATCC 53582 strain was used for the production of BC. All reagents used were of analytical quality and were purchased from Sigma-Aldrich (Poland) or Chempur (Poland).

### Experiment

#### Bacterial Cellulose Beads Preparation

The culture of *K. xylinus* was carried out in a 25-ml Erlenmeyer flask with Herstin-Schramm (HS) medium containing glucose 20 g/l, yeast extract 2.0 g/l, peptone 2 g/l, citric acid 1.15 g/l, Na_2_HPO_4_ 2.7 g/l, and MgSO_4_ ∙7H_2_O 0.06 g/l with 1% ethanol. Prepared medium was inoculated with 1 ml of 2-week-old starter culture. The cultivation was carried out at 28 °C, on a laboratory shaker at 180 rpm for 24 h. After this time, the formed “spheres,” hereinafter referred to as “beads” of BC (BCB), were picked up using a laboratory strainer and rinsed in deionized H_2_O (dH_2_O) to remove culture medium. The BCB was then digested with 0.1 M NaOH at 80 °C for 30 min (3×) to remove bacterial cells and residual nutrient components. Finally, the cellulose was rinsed again with dH_2_O until the pH stabilization at 7.0. Cellulose beads prepared in this way were stored at 4 °C until use.

#### Modification of Bacterial Cellulose Beads and Lecitase® Ultra Immobilization

**Fig. 1:**
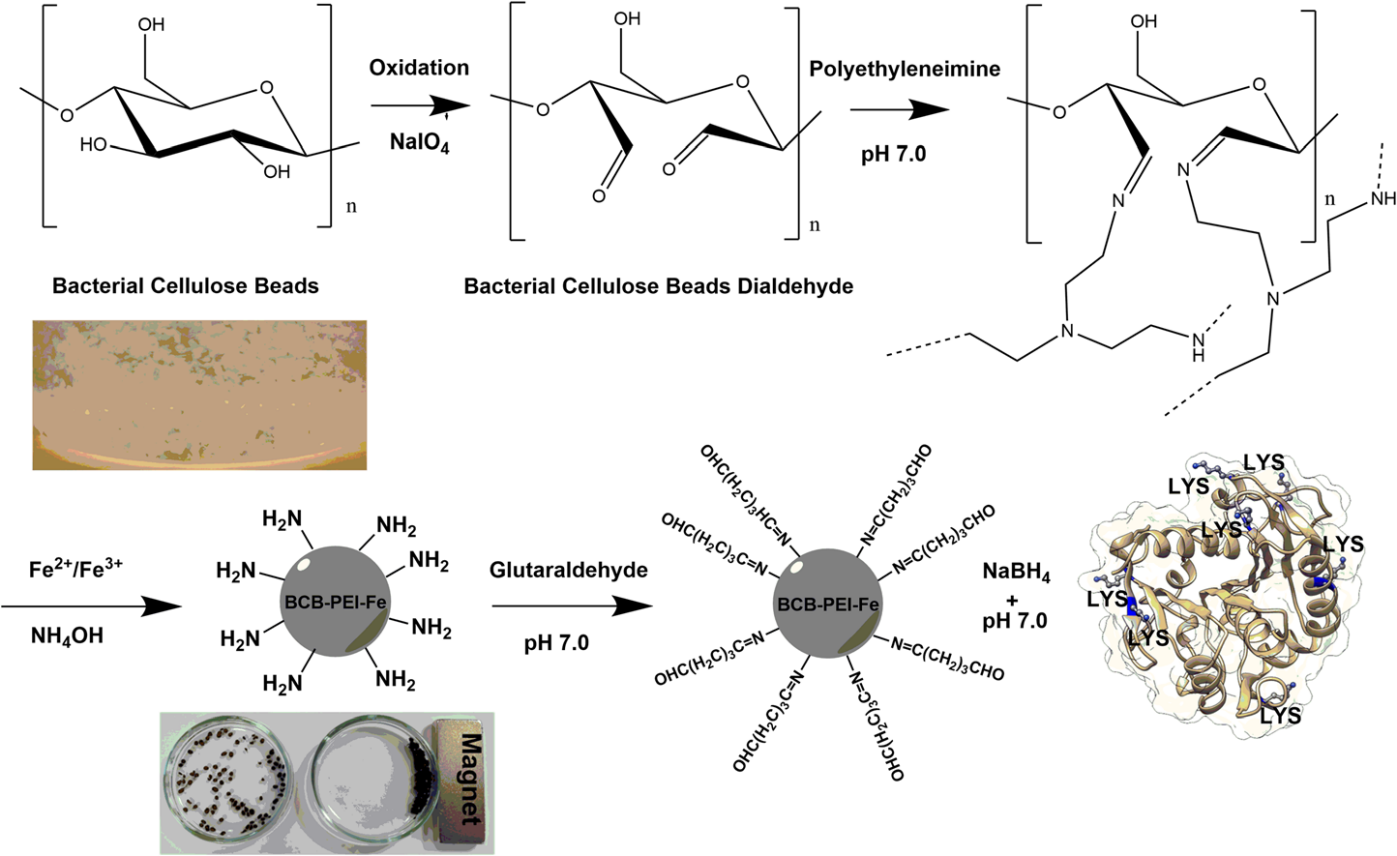
The illustration of carrier preparation and Lecitase® Ultra immobilization process

The preparation of the carrier was carried out according to optimized protocol (Fig. 1). In the first stage, the beads were oxidized using sodium periodate (NaIO_4_). The fixed mass of BCB was transferred to the plastic tubes, added in a ratio of 1:2 with 100 mM aqueous NaIO_4_ solution and mixed on a roller shaker for 4 h at 25 °C. The oxidized BCB was then rinsed with dH_2_O and 1% (*v*/*v*) polyethyleneimine (PEI) solution (*M*_*w*_ 750,000) in 100 mM phosphate buffer at pH 7.0 was added in a 1:1 ratio and further incubated on a roller mixer for 12 h at 25 °C. Obtained BCB saturated with PEI (BCB-PEI) was washed with 100 mM phosphate buffer at pH 7.0 for removing excess of unbounded PEI. In the next step, BCB-PEI was saturated with the mixture of Fe^2+^/Fe^3+^ ions (sulfate salts) in molar ratio 1:2 by addition of 1 volume of beads to 3 volumes of previously prepared Fe^2+^/Fe^3+^ solution and mixed on a roller shaker for 20 min at room temperature. After aspiration of Fe^2+^/Fe^3+^ solution, 3 volumes of 2.5% NH_4_OH aqueous solution were added and incubated for 30 min at 60 °C with periodic stirring. Finally, the obtained BCB-PEI-Fe was rinsed with dH_2_O until the pH stabilizes and stored in water at 4 °C until further use.

#### Enzyme Immobilization

Initially, the BCB-PEI-Fe were activated with 1% glutaraldehyde in 100 mM phosphate buffer at pH 7.0 by adding 2 volumes of solution in relation to the BC volume and mixed using a roller shaker at room temperature for 1 h. The activated BCB-PEI-Fe was rinsed three times in 100 mM phosphate buffer of pH 7.0 with 100 mM NaCl to remove unbound glutaraldehyde. Immobilization of the enzyme was performed by adding 2 volumes of the enzyme solution to 1 volume of BCB-PEI-Fe activated with glutaraldehyde and incubation at 4 °C for 24 h. After the incubation, the supernatant was removed, and the obtained matrix was washed twice with 100 mM phosphate buffer pH 7.0. Then, 4 volumes of sodium borohydride (1 mg/ml in 100 mM phosphate buffer of pH 7.0) solution were added to the immobilized enzyme and incubated at 4 °C for 1 h. In the next stage, BCB-PEI-Fe was rinsed once in 100 mM phosphate buffer of pH 7.0 containing 100 mM NaCl and 0.25% Triton-X100, and then twice in 100 mM phosphate buffer of pH 7.0.

#### Determination of the Activity of the Native and Immobilized Enzyme

As substrate for the determination of LU activity, 4-nitrophenol palmitate (pNPP) was used at a concentration of 0.5 mM in Tris-Cl 50 mM pH 8.5 in the presence of 0.25% Triton X-100. The stock solution of pNPP (3.3 mg per ml) was prepared in 2-propanol, mixed with 10 volumes of assay buffer, and heated to 60 °C for 15 min to obtain transparent solution. Activity was determined by measuring the absorbance changes for 5 min at 30 °C at wavelength λ = 348 nm (ε = 5.400 mM^−1^ cm^−1^) using a microplate reader. One Lecitase® Ultra unit releases 1 μmol pNPP per minute. The specific activity of the enzyme activities was expressed as units per mg protein, immobilized enzyme as units per g of wet weight of the carrier.

#### Protein Concentration Determination

Protein concentrations were assayed by Bradford method with bovine serum albumin as a standard [22].

#### Efficiency of Binding of the Enzyme to the Carrier

In order to test the ability of binding the enzyme with the carrier, Lecitase® Ultra formulation was diluted in phosphate buffer of pH 7.0 in the range of activity of 500 to 1200 mU/ml and protein concentration range of 0.45 to 1.8 mg/ml. The prepared dilutions were then mixed with the activated carrier. Next, prepared samples were incubated overnight at 4 °C. After this time, activity of LU was measured in each of the trials according to the methodology given above.

The yield of immobilization was calculated from the equation:

$$ \mathrm{Yield}\left(\%\right)=100\kern0.5em \frac{\mathrm{immobilized}\kern0.5em \mathrm{activity}}{\mathrm{starting}\kern0.5em \mathrm{activity}} $$

where immobilized activity is the difference between starting and remaining activity in the binding solution [23].

#### Optimum pH of Free and Immobilized Enzymes

In order to check the pH optimum for free and immobilized enzymes, the enzyme activity was measured at pH 6.0, 6.5, 7.0, 7.5 (50 mM phosphate buffer), and at pH 8.0, 8.5, and 9.0 (50 mM Tris-Cl buffer).

#### Temperature Optimum of Free and Immobilized Enzymes

The temperature optimum of the free enzyme was measured at 25, 30, 35, 40, 45, 50, 55, and 60 °C. Before adding the enzyme, substrate solution was equilibrated and next 10 ml of the enzyme solution in 50 mM phosphate buffer of pH 7.5 to 300 μl of substrate and incubated for 5 min at suitable temperatures. Determination of the optimum temperature of the immobilized enzyme was carried out by transferring 300 μl of the heated substrate to the tube with buffer containing ~25 mg of the immobilized enzyme. The mixture was then incubated for 5 min at appropriate temperatures. The activity was expressed in relative terms taking the highest activity at a given temperature for 100%.

#### Thermal Stability of Free and Immobilized Enzymes

The thermal stability of the free and immobilized enzymes was determined at a selected temperature 40, 50, and 60 °C by incubation in 1 ml of 50 mM phosphate buffer pH 7.5 for 10–60 min. The residual activity of free and immobilized enzyme was expressed in relative terms taking initial activity as 100%.

#### Determination of Kinetic Parameters Free and Immobilized Lecitase® Ultra

Kinetic parameters K_M_ and V_max_ of immobilized and free LU were determined by measuring the rate of hydrolysis of pNPP. Initial velocities were determined for substrate concentrations in the range from 0.05 to 1.5 mM in 50 mM Tris-Cl of pH 8.5 in the presence of 0.25% Triton X-100. The kinetic constants were determined according to the Michaelis-Menten kinetics model using a non-linear regression model using the Origin8pro program.

### Operating Parameters

#### Effect of Reusability on Immobilized Enzyme Stability

The reusability of immobilized enzyme was determined by using the immobilized beads for 10 times. After each cycle of reaction, the BCB-PEI-Fe beads were removed and washed with phosphate buffer 100 mM (pH 7.5) to clean it from residual substrate and products of reaction from immobilized beads. Next, the immobilized beads were transferred into fresh reaction medium to start reaction. The initial activity was considered as 100%.

#### Determination of Storage Stability Immobilized Lecitase® Ultra

The immobilized enzyme was stored as suspension in 50 mM phosphate buffer of pH 7.0, and activity was determined several times during 28 days of storage at 4 °C. At this time, an equal amount of carrier with immobilized enzyme was collected every 2–3 days and its activity was measured. The initial activity was considered as 100%.

### Carrier Property Determination

#### Scanning Electron Microscopy

Scanning electron microscopy (SEM) was performed using a high-resolution field emission gun scanning electron microscope (ZEISS EVO MA 25, Oberkochen, Germany). The samples of modified BCB were firstly fixed by 3% glutaraldehyde solution in phosphate buffer of pH 7.0 by 30 min. Next, samples were flushed by deionized water to remove extended amount of glutaraldehyde. Then, fixed BCB were dehydrated in graded series of ethanol dilution 10–100% (5 min each) and finally dried with the hexamethyldisilazane (HMDS) chemical drying series, ethanol:HMDS 1:1, 2:1, and 100% HMDS two times 20 min and allow to air dry overnight under a fume hood. Afterwards, prepared beads were used to characterize the morphology of the functionalized BCB. Prior to the SEM, all the samples were fixed onto SEM by the sputtering with Au/Pd (60:40) using Q150R ES device (Quorum Technologies, Laughton, UK).

#### Attenuated Total Reflectance Fourier Transform Infrared Spectral Studies of Modified Cellulose Beads

Samples before attenuated total reflectance Fourier transform infrared (ATR-FTIR) analysis were dried at room temperature for 24 h. The analysis was carried out using a Bruker spectrophotometer with an ATR-FTIR adapter. The spectra were collected in the range of 4000–400 cm^−1^ with a resolution of 8 cm^−1^ (64 scans). The obtained ATR-FTIR spectra were analyzed using the Omnics and Origin8pro software.

## Results and Discussion

### Carrier Properties

**Fig. 2:**
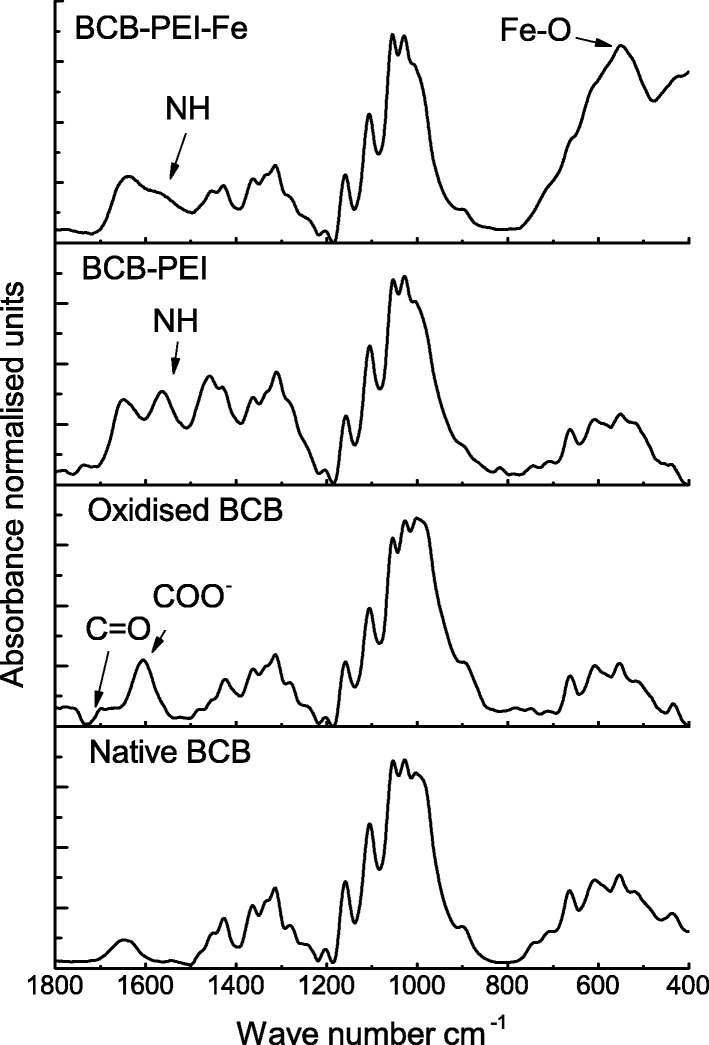
The ATR-FTIR spectra of BCB from subsequent stages of modification. The spectra were restricted to the region from 1800 to 400 cm^−1^ and normalized to 1 at 1060 cm^−1^

Analysis of the ATR-FTIR spectrum of modified BC indicates its effective modification by PEI and Fe_3_O_4_. Oxidation step using sodium periodate increased the intensity of the bands from 1600 to 1780 cm^−1^ (Fig. 2). The strands belonging to the C–O stretching vibration of carboxylic group are usually visible in this region (the most intense ones at 1610 cm^−1^). Sodium periodate opens the glucose pyranose ring by specific cleaving the C2-C3 bond. It leads to formation of two aldehyde groups, the specific C=O stretches of which may be detected by the presence of 1725-cm^−1^ peak [24]. After oxidation of BCB-PEI, a peak at 1560 cm^−1^, which is appropriate for NH_2_ in bend plane, appears. Further modification of BCB-PEI by saturation with Fe_3_O_4_ leads to the spectrum modification in the range from 700 to 500 cm^−1^, where band at 585 cm^−1^ shows Fe–O stretching vibrations typical for magnetite particles. Formation of magnetite particles was also confirmed by XRD analysis (Fig. S1). Above-mentioned changes confirm successful introduction of functional groups to BCB structure. In turn, differences in 1200- to 950-cm^−1^ spectrum range indicate alterations of crystalline structure of cellulose microfibrils. Reduction of the intensity of bands in the range from 1000 to 900 cm^−1^, marking the conformation of the primary alcohols present in the glucose structure [25], indicates an increase in the amount of amorphous cellulose fraction compared to crystalline one, which may affect the mechanical properties of the obtained carrier [26]. At the stage of oxidation, the intensity of bands did not differ significantly from native BC. However, Guo et al. [27] and several other authors reported a decrease in the cellulose crystallinity due to the periodate oxidation. This can be explained by relatively short oxidation time and low concentration of sodium periodate applied (100 mM, 4 h). These conditions were, however, well suited for permanent binding of PEI by BCB. Modification with PEI had the greatest impact on the BCB microfibril structure, for which the reduction in the intensity of bands at 1003 and 980 cm^−1^ is the most pronounced and does not change after next modification. Further BCB-PEI modification by introducing Fe_3_O_4_ into the carrier structure resulted in the decrease of band intensity at 1560 cm^−1^, indicating lower content of amino groups on the surface of BCB-PEI-Fe.

### SEM Morphology of Modified BCB

**Fig. 3:**
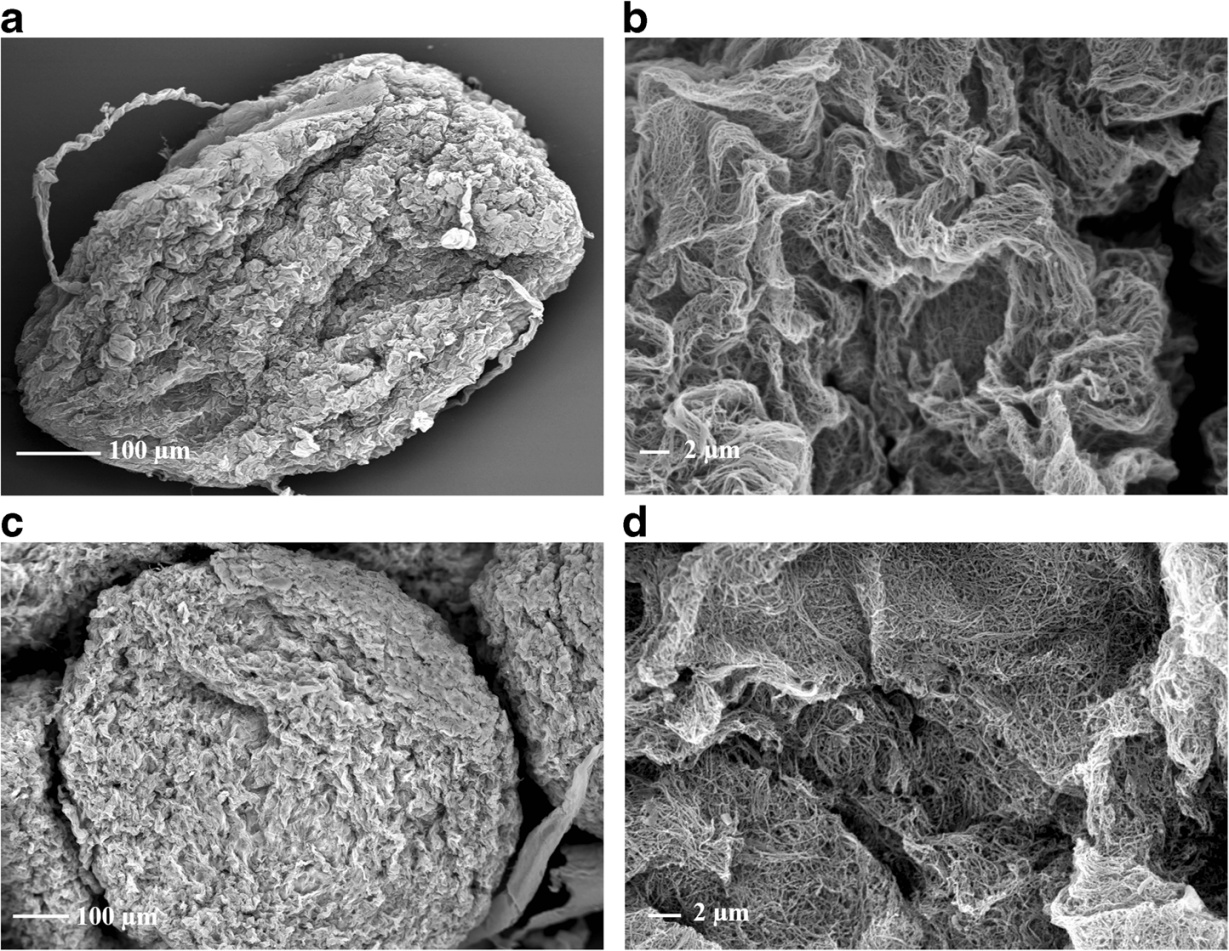
SEM images of unmodified BCB (**a**, **b**; magnification ×300 and ×7000) and BCB-PEI-Fe with immobilized Lecitase® Ultra (**c**, **d**; magnification ×300 and ×7000)

The SEM pictures show the structure of native and modified BCB (Fig. 3). Pictures of modified and native spheres of BC revealed strongly corrugated surface formed by layers of randomly oriented cellulose microfibrils. As for BCB-PEI-Fe, SEM analysis revealed presence of Fe_3_O_4_ particles associated with microfibrils evenly distributed throughout the structure. Moreover, BCB-PEI-Fe displayed high porosity of BCB, and it significantly increased the area available for immobilization and might also affect the catalytic properties of the immobilized Lecitase® Ultra.

### Effectiveness of Immobilization Process on Modified BCB

**Fig. 4:**
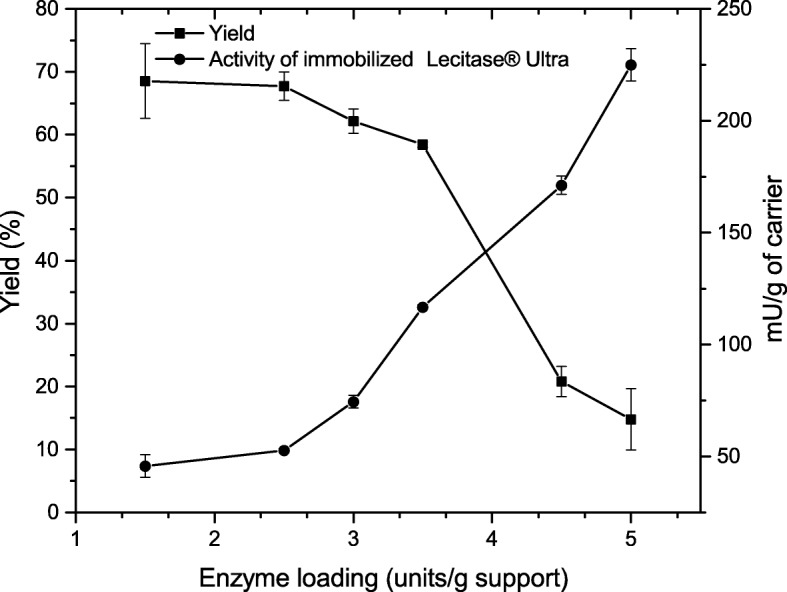
The yield (%) of immobilization process and activity of immobilized Lecitase® Ultra depending on the enzyme solution initial activity used to immobilization

Values of obtained yield indicate that modified BCB displays high ability to bind the enzyme (Fig. 4). The immobilized enzyme activity in BCB-PEI-Fe showed positive linear relation to the enzyme initial activity loading, in contrast to the percent of immobilization yield value. The highest specific activity of Lecitase® Ultra, 225.0 ± 7.1 mU/g, was obtained for the initial activity of the preparation used to immobilize 5.0 U/g of support with yield being only 14.8 ± 7.0%. In the range of the initial loading activity of LU from 1.5 to 3.0 U/g, the yield obtained did not exceed 70%; however, specific activity was relatively low and was within the range of 74.5 ± 3.0 to 45.7 ± 5.0 mU/g. The most favorable ratio between loading activity and yield of immobilization was found for initial units loading of 3.5 U/g with final specific activity of immobilized LU equal to 116.2 ± 1.0 mU/g and yield 58.5 ± 0.4%.

### Optimum pH for Immobilized Lecitase® Ultra

**Fig. 5:**
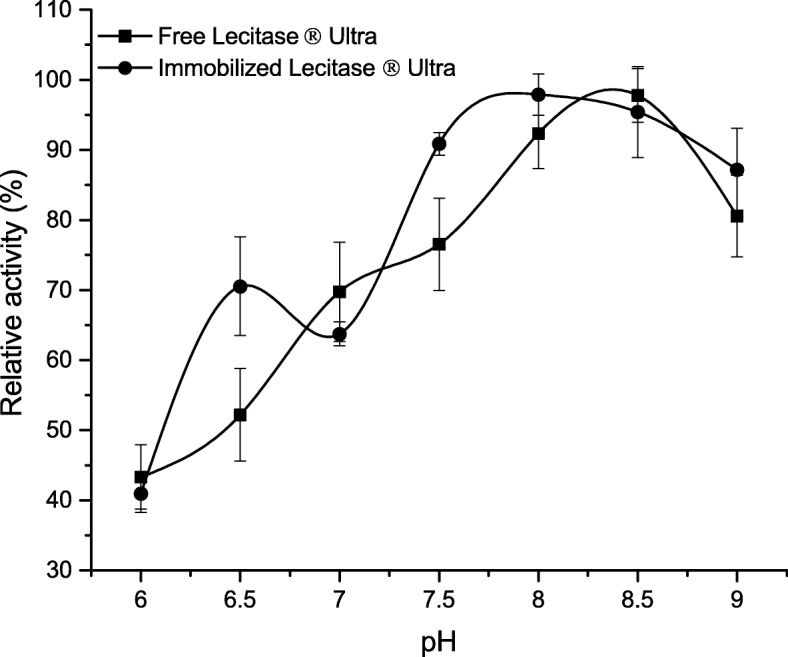
Effect of pH on activity of free and immobilized on BCB-PEI-Fe Lecitase® Ultra

The effect of pH on the immobilized enzyme was analyzed within the pH range of 6.0–9.0 (Fig. 5). Optimum pH value for free and immobilized LU was 8.5. This value is consistent with the optimum reported by Mishra et al. [28], who also studied the effect of pH on Lecitase® Ultra activity, using pNPP as a substrate. Free and immobilized enzymes showed similar pH optimum profile, with no significant differences in the entire analyzed range. Lecitase® Ultra entrapped in gelatin cross-linked with glutaraldehyde showed significant shift at a pH optimum of up to 7.5 in comparison to free enzyme, for which the pH optimum was 6.5–7.0. On the other hand, dos Santos et al. [29] showed that depending on the type of functional groups present on the surface of the carrier and the immobilization method, the pH optimum of the LU profiles ranges from 6.0 to 9.0 and the pH activity profile also varies. Lecitase® Ultra immobilized on epoxy-activated polymer DILBEAD-VWR modified by polyethyleneimine showed a shift of the pH optimum towards the alkaline reaction, probably due to the presence of amino groups on the carrier surface [30]. Wu et al. [12] analyzed lipases other than Lecitase® Ultra (i.e., *Candida rugosa*-derived lipases) and immobilized these enzymes on BC disks. Such immobilized enzymes displayed optimal activity at pH equal to 7.0, analogously to unbound enzyme. Contrary results were obtained by Huang et al. [31], who reported that pH optimum for free *C. rugosa*-derived lipase is 5.5, while immobilized enzyme displayed optimum at 6.1. The lack of changes in the optimum pH of the immobilized LU presented in our study can be a result of the immobilization process itself, in which the spacer (glutaraldehyde) used allows the bound enzyme to be removed from the support surface. This probably prevents from the influence of local changes in the H^+^ and OH^−^ gradient on the immobilized enzyme.

### Influence of Temperature on Activity and Stability of Immobilized Lecitase® Ultra

**Fig. 6:**
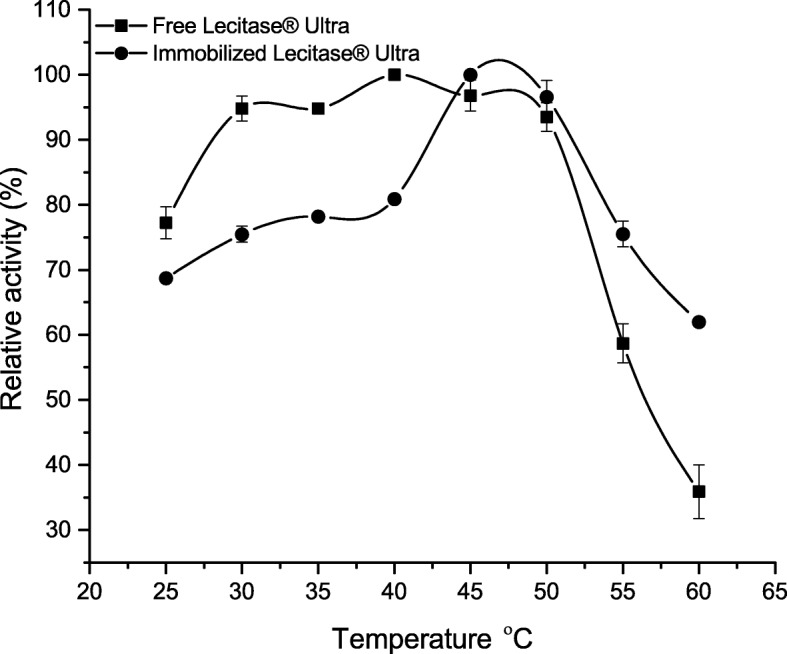
Effect of temperature on the activity of the free and immobilized on BCB-PEI-Fe Lecitase® Ultra

The activity of immobilized and free enzyme was analyzed in the temperature range from 25 to 60 °C (Fig. 6). The optimal temperature for the free form of enzyme was 40 °C. Free enzyme retained high activity up to 50 °C, and application of 50–60 °C led to sharp drop of lipase activity. Optimal temperature for immobilized enzyme was in the range of 45–50 °C. It should be noted that application of higher temperatures, i.e., 50–60 °C, led to lower drop of immobilized enzyme stability in comparison to free lipase. Lecitase® Ultra trapped in gelatin also showed a shift of the optimum temperature from 40 to 50 °C [32]. Similar results were obtained by Zhan et al. [33]; however, this shift was observed when 50–60 °C temperature was applied. Lipase from *Candida cylindracea* immobilized on magnetic poly(methacrylate-divinylbenzene) microsphere also showed a shift in the temperature optimum from 37 to 50 °C [34]. Contrary to this, LU immobilized on triacetate cellulose showed similar optimum temperature to the free enzyme (40 °C) [35]. Immobilization process also did not significantly affect the temperature profile of the lipase derived from *C. rugosa* [12]. The shift of the catalytic temperature optimum of immobilized enzymes is a frequent phenomenon resulting from the decrease of flexibility of immobilized protein [36]. The impact of the immobilization process depends on the method of immobilization and resulting interaction between the carrier and the enzyme. The loss in flexibility, which is necessary to obtain the proper conformation of the catalytic region, enforces the need to partially relax the resulting bonds between the carrier and the enzyme and manifested by higher-temperature optima of immobilized enzymes. Immobilization of LU on BCB-PEI-Fe, by introducing a spacer in the form of glutaraldehyde, allows to distance the enzyme from the surface of the carrier itself. As a result, it is possible to retain comparable flexibility to the free form of the enzyme, which could be the reason for similar values of temperature optima for free and immobilized enzymes.

**Fig. 7:**
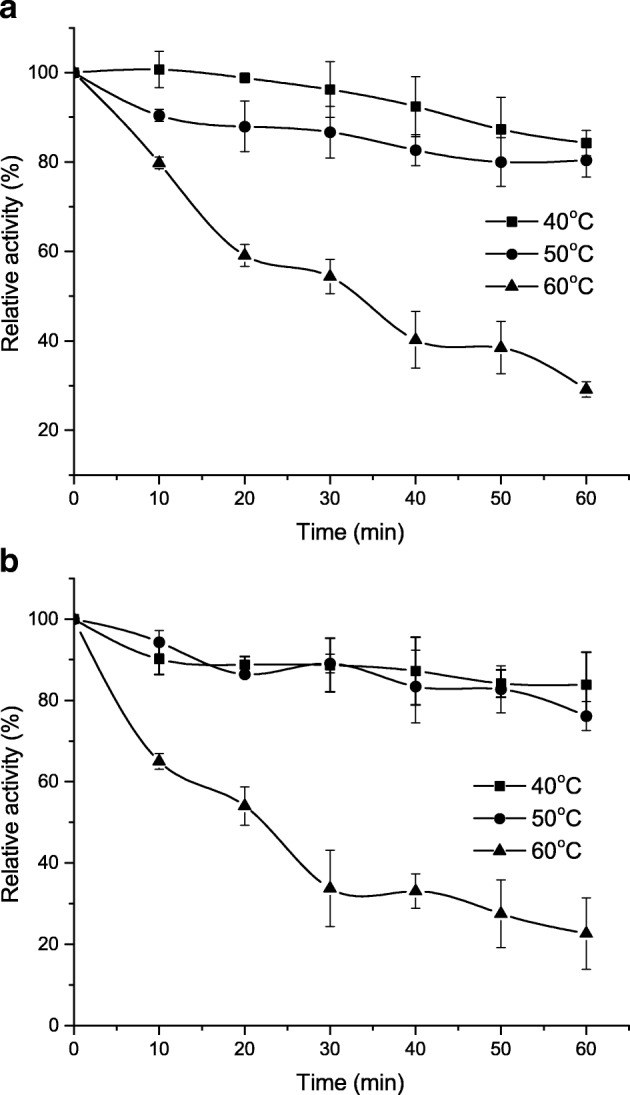
Effect of temperature on stability of free (**a**) and immobilized on BCB-PEI-Fe Lecitase® Ultra (**b**)

Both free and immobilized enzymes showed similar temperature stability profile (Fig. 7a, b). At 40 and 50 °C, after 1 h of incubation, the native as well as immobilized enzymes retained over 80% of the activity. However, at 60 °C, after just 20 min of incubation, both free and immobilized enzymes lost more than 40% of its initial activity. The immobilization process has usually a positive effect on the enzyme stability profile increasing its resistance to higher temperatures. For example, LU entrapped in gelatin maintained 60% of its initial activity at 50 °C compared to 30% for the free form of the enzyme [32]**.** Immobilization of LU on hydrophobic polystyrene macroporous resin (DA-201) also resulted in a significant increase in the enzyme temperature stability at 55 °C, retaining over 60% of its initial activity [36]**.** The immobilization of LU to cellulose triacetate also increased the temperature stability of the enzyme, especially at 50 and 60 °C [36]. However, immobilization of Lecitase® Ultra on epoxy-activated polymer (DILBEAD-VWR) did not significantly affect the stability of the enzyme at high temperature [30]. The rationale for the increase in thermal stability of immobilized enzymes observed by majority of researchers is drop of these proteins’ flexibility, i.e., their ability to undergo changes in molecular structure. This is direct result of enzyme immobilization and may also have an impact on their activity. However, depending on the immobilization method and the type of interactions affecting the binding of the enzyme to the carrier, the effect of immobilization on the enzyme structure may be different and, as many examples show, immobilization does not always lead to increased thermal stability of the enzyme [37]**.**

### Effect of Immobilization on Catalytic Constants of Lecitase® Ultra

Immobilization of enzymes often leads to disturbances in mass exchange leading to hindered access of the enzyme to the substrate. It frequently manifests in an increase in K_M_ value of the immobilized enzyme comparing to the free form. Bacterial cellulose, as indicated by SEM analyses, is a material with extensive molecular structure characterized by high porosity. Therefore, significant increase in K_M_ value for the immobilized enzyme could be expected due to diffusion of the substrate to the interior of the carrier. Immobilized on BCB-PEI-Fe, LU showed similar K_M_ value to the free form of the enzyme (Table 1). It may be probably related to the binding of the enzyme by the carrier mainly on its surface, which significantly reduces the probability of disturbances in the mass transfer process. However, catalytic efficiency (Kcat/K_M_) calculated for immobilized enzyme was almost fourfold lower in comparison to free LU. Lecitase® Ultra immobilized onto a polystyrene DA-201 resin was characterized by ≈25% increase in K_M_ value (tributyrin as a substrate) as compared to the free form of the enzyme, suggesting the influence of immobilization process on mass transfer [36]. Apart from possible difficulties in binding of substrate associated with the structure of the carrier as reported by Fernandez-Lorente et al. [38], changes in the K_M_ constant reflecting the degree of affinity of the enzyme for the substrate may also depend on possible conformational changes of the enzyme resulting from binding to the support. Depending on the type of functional groups present on the surface of the carrier, the strength and type of interaction with the enzyme is different and can influence on the 3D structure of the immobilized LU in various ways [39]. This can lead to the reduction in turnover number (K_cat_) of the immobilized enzyme as well as its substrate specificity [40]. However, observed reduction in catalytic efficiency of immobilized LU can be compensated by the possibility of repeated use of this form of enzyme.

**Table 1:** Influence of immobilization process on K_M_ and V_max_ and catalytic efficiency K_cat_/K_M_ of Lecitase® Ultra

	V_max_ (μmol l^−1^ min^−1^)	K_M_ (mM)	K_cat_ × 10^3^ (s^−1^)	K_cat_/K_M_ (M^−1^ s^−1^)
Immobilized LU	8.7 ± 0.7	0.40 ± 0.07	1.9 ± 0.15	4.8 ± 0.8
Free LU	49.6 ± 4.7	0.45 ± 0.05	9.3 ± 0.9	20.9 ± 2.3

The data shown are representative of three independent experiments (mean ± SD)

^a^Determination of catalytic constants was done with use 10 μg of free enzyme and 25 mg of immobilized enzyme that contained 70 μg of protein

### Reusability of Immobilized Lecitase® Ultra

**Fig. 8:**
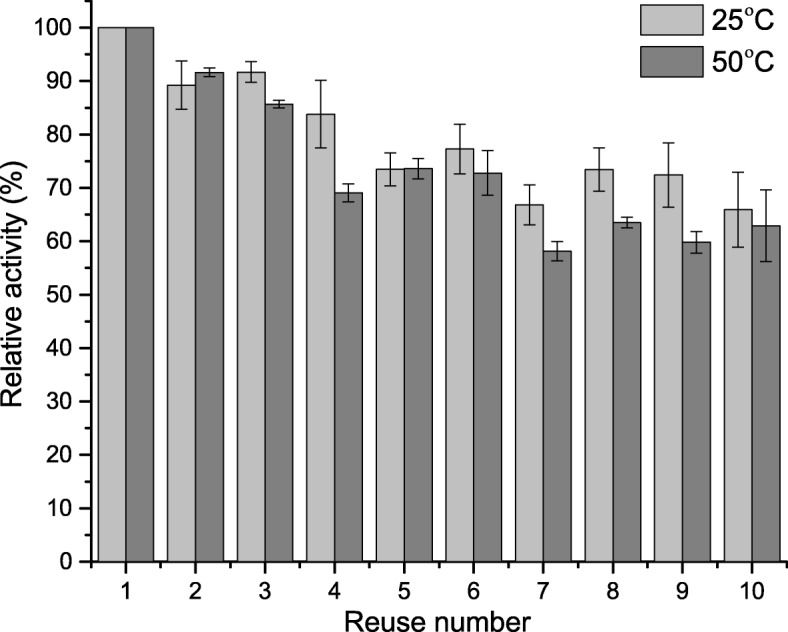
Reusability of immobilized on BCB-PEI-Fe Lecitase® Ultra. The immobilized enzyme was tested at room temperature (25 °C) and on optimal temperature (50 °C)

One of the most important features of an immobilized enzyme, with regard to its industrial application, is the possibility of its repeated use without significant loss of activity. Analysis of aforementioned parameter indicated high stability of the immobilized LU (Fig. 8). After 10 cycles, the immobilized enzyme retained significant activity, reaching 70% of the initial activity at both tested temperatures (25 and 70 °C). LU immobilized on triacetate cellulose maintained less than 20% of the initial activity as well as trapped in gelatin after 5 cycles. The reason for such low reusability was the possibility of enzyme leakage from gelatin matrix and in the case of immobilization on triacetate cellulose, the presence of Triton-X-100 in the reaction mixture, which significantly enhanced the enzyme desorption from the carrier [32, 36]. Despite the presence of mentioned detergent in the reaction mixture used to determine the activity, resulting covalent bond between the carrier and enzyme effectively prevents from LU desorption from the BCB-PEI-Fe. The decrease in the activity of immobilized LU observed in successive cycles was probably related to the denaturation of parts of enzyme molecules rather than rinsing of the enzyme from the carrier, because activity of LU in washing buffer was not detected. Wu et al. [12], using the BC in the form of glutaraldehyde modifying membranes as a carrier for the immobilization of lipase from *C. rugosa* and different method of drying, showed significant effect of these factors on reusability of the immobilized enzyme. In optimal conditions, the lipase immobilized by these authors retained nearly 60% of its initial activity after 15 cycles. Values of reusability, obtained in the current study, indicate high operational stability of the immobilized LU on BCB-PEI-Fe and its significant resistance to repeated use, which is of fundamental importance in the further use of the immobilized enzyme.

### Storage Stability of Immobilized Lecitase® Ultra

**Fig. 9:**
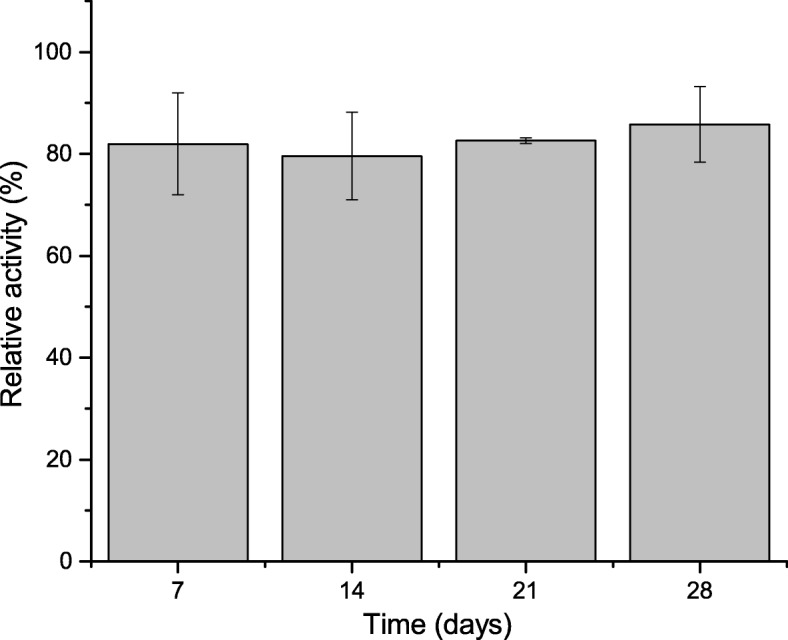
Effect of storage time on activity of immobilized on BCB-PEI-Fe Lecitase® Ultra

In addition to the reuse, it is also important to store the immobilized enzyme for an extended period of time without significant decrease in its activity. In order to check this parameter, the activity of the immobilized enzyme was measured at various intervals for 28 days, when the enzyme was stored at 4 °C. After 7 days, the activity of the immobilized enzyme decreased by about 20%; however, the activity remained unchanged for the next 3 weeks (Fig. 9). Huang et al. [31] immobilized lipase from *C. rugosa* on electrospun cellulose nanofiber membrane, and as indicated by this author, the enzyme retained 60% of the initial activity after 30 days of storage. Liu et al. [36] immobilized lipase produced by *C. rugosa* on Fe_3_O_4_ nanoparticles. The authors showed that the enzyme retained 70% of its initial activity after 30 days of storage. Kuo et al. [41] used the same lipase, Fe_3_O_4_ saturated chitosan as a carrier for immobilization, and after 13 days of storage at 25 °C, the enzyme retained 100% of the initial activity. Li et al. [42] stored lipase produced by *C. rugosa* and immobilized on polyacrylonitrile for 20 days; after that time, the enzyme retained 95% of its initial activity. Analysis of enzyme stability during storage performed in the current study showed that immobilized on BCB-PEI-Fe, it retains the level of its activity for at least 28 days.

Effective immobilization of Lecitase® Ultra on functionalized spheres of bacterial cellulose extends the possibility of its use in various types of bioprocesses. Moreover, the immobilized LU on the carrier sensitive to the external magnetic field has the main advantage of being easily separable from the reaction medium. As a result, utilizing these obvious benefits, the enzyme immobilized on this type of carrier can be used in processes carried out using bioreactors supported with various types of magnetic field [43]. In this type of reactors, the use of carriers’ sensitivity to magnetic field gives the opportunity of mixing without use of traditional mechanical stirrers, thus increasing the stability of the carrier due to minimizing the mechanical shear, while mixing and also improving the mass transfer [44]. One of the frequent uses of lipases and also LU is their ability to synthesize various types of esters that can be used as biofuels or precursors of substances desired in the pharmaceutical or cosmetic industries. The utilization of magnetic field in processes catalyzed by immobilized enzymes can potentially affect the reaction mode and in this way giving the possibility of obtaining new compounds or increasing the process effectiveness [45].

## Conclusions

Bacterial cellulose beads modified by polyethyleneimine and ferromagnetic material were used as a new support material for the immobilization of Lecitase® Ultra. The important advantage of using a natural biopolymer as BC is also significant reduction in costs associated with its purification and preparation of the carrier for further modification. Properties of this new carrier allow for efficient immobilization of analyzed enzyme and also simplification during its manipulation by easy separation form reaction medium with use a regular magnet only. Immobilization process did not significantly influence on main catalytic parameters of LU (pH, temperature optima). However, immobilized Lecitase® Ultra showed fourfold lower catalytic efficiency, which on the other hand was compensated by high resistance to reuse.

The resulting carrier is characterized by unique properties allowing its further modification to be used for the immobilization of many other enzymes that can be applied in biotechnological process, in which magnetic field is applied as a force for a biocatalyst separation and modification of its properties.

## Electronic Supplementary Material

ESM 1 (DOCX 86 kb)
