# Supplementary material for: Functionalized Magnetic Bacterial Cellulose Beads as Carrier for Lecitase® Ultra Immobilization
Source: Appl Biochem Biotechnol. 2018 Jun 18;187(1):176–93. doi: 10.1007/s12010-018-2816-1 (PMC6326999; doi:10.1007/s12010-018-2816-1)
Supplement: Supplementary file 1 — (DOCX 86 kb) [file 12010_2018_2816_MOESM1_ESM.docx]

**S1.** X-ray diffraction (XRD) analysis was performed on a ULTIMA IV/Rigaku/2008 X-ray diffractometer with Cu Kα radiation (λ = 1.5418 Ǻ). The 2θ range applied in the measurement was from 10° to 70°.


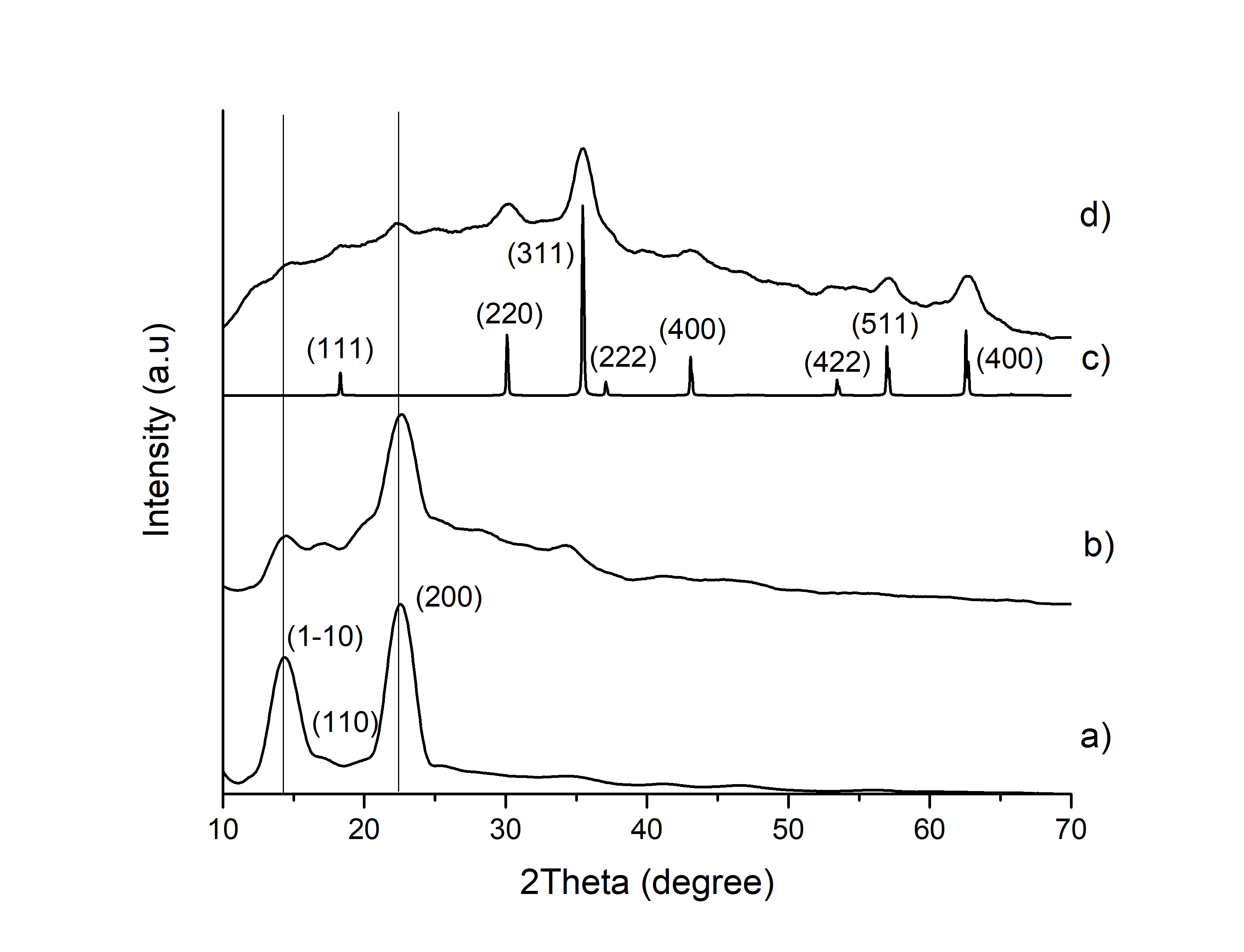


**Fig. S1.** The XRD pattern for non-modified (a), oxidized and PEI saturated bacterial cellulose beads (b), Fe_3_O_4_ diffraction pattern from the International Centre for Diffraction Data® (ICDD®) PDF+ 2018 database card No. 01-080-6402 (c) and bacterial cellulose beads modified by PEI and Fe_3_O_4_ (d).

The native bacterial cellulose XRD diffraction spectra showed three characteristic diffraction peaks at 2θ 14.5° (101), 16.6° (110), and 22.7° (200) for high crystalline BC (a). Modification of BCB through oxidation and further saturation by PEI alter intensity of the aforementioned diffraction peaks. This indicates the change in bacterial cellulose crystallinity to more amorphous phase. The introduction of Fe_3_O_4_ to the structure of modified bacterial cellulose beads results in an appearance typical for magnetite diffraction peaks at 2θ 18.2° (111), 30.27° (220), 35.53° (311), 43.26° (400), 53.11° (422), 56.88° (511) and 62.76° (440) (ICDD® PDF+ 2018 card No. 01-080-6402). Entrapping of Fe_3_O_4_ particles in BCB matrix did not result in its phase change. The intensity of diffraction peaks characteristic for bacterial cellulose was reduced by excessive amount of Fe_3_O_4_ entrapped in BCB matrix.

**Reference**

S. Park, J. O. Baker, M. E. Himmel, P. A. Parilla, D. K. Johnson, Cellulose crystallinity index: measurement techniques and their impact on interpreting cellulase performance, Biotechnol. Biofuels. 24 (2010) 3-10.

V.A.J. Silva, P.L. Andrade, M.P.C. Silva, A. D. Bustamante, Luis De Los Santos Valladares, J. Albino Aguiar, Synthesis and characterization of Fe_3_O_4_ nanoparticles coated with fucan polysaccharides, J. Magn. Magn. Mate, 343 (2013) 138-143.

R. Drozd, R. Rakoczy, M. Konopacki, A. Frąckowiak, K. Fijałkowski, Evaluation of usefulness of 2DCorr technique in assessing physicochemical properties of bacterial cellulose, Carbohydr. Polym. 161 (2017) 208-218.
